# Ex vitro hairy root induction in detached peanut leaves for plant–nematode interaction studies

**DOI:** 10.1186/s13007-017-0176-4

**Published:** 2017-04-11

**Authors:** Larissa Arrais Guimaraes, Bruna Medeiros Pereira, Ana Claudia Guerra Araujo, Patricia Messenberg Guimaraes, Ana Cristina Miranda Brasileiro

**Affiliations:** 1 Parque Estação Biológica, Embrapa Recursos Genéticos e Biotecnologia, CP 02372, Final W5 Norte, Brasília, DF Brazil; 2 Universidade de Brasília, Campus Darcy Ribeiro, Brasília, DF Brazil

**Keywords:** *Meloidogyne*, *Arachis*, *Agrobacterium rhizogenes*, Genetic transformation, Expansin like-B

## Abstract

**Background:**

Peanut (*Arachis hypogaea*) production is largely affected by a variety of abiotic and biotic stresses, including the root-knot nematode (RKN) *Meloidogyne arenaria* that causes yield losses worldwide. Transcriptome studies of wild *Arachis* species, which harbor resistance to a number of pests and diseases, disclosed several candidate genes for *M. arenaria* resistance. Peanut is recalcitrant to genetic transformation, so the use of *Agrobacterium rhizogenes*-derived hairy roots emerged as an alternative for in-root functional characterization of these candidate genes.

**Results:**

The present report describes an ex vitro methodology for hairy root induction in detached leaves based on the well-known ability of peanut to produce roots spontaneously from its petiole, which can be maintained for extended periods under high-humidity conditions. Thirty days after infection with the *A. rhizogenes* ‘K599’ strain, 90% of the detached leaves developed transgenic hairy roots with 5 cm of length in average, which were then inoculated with *M. arenaria*. For improved results, plant transformation, and nematode inoculation parameters were adjusted, such as bacterial cell density and growth stage; moist chamber conditions and nematode inoculum concentration. Using this methodology, a candidate gene for nematode resistance, *AdEXLB8,* was successfully overexpressed in hairy roots of the nematode-susceptible peanut cultivar ‘Runner’, resulting in 98% reduction in the number of galls and egg masses compared to the control, 60 days after *M. arenaria* infection.

**Conclusions:**

This methodology proved to be more practical and cost-effective for functional validation of peanut candidate genes than in vitro and composite plant approaches, as it requires less space, reduces analysis costs and displays high transformation efficiency. The reduction in the number of RKN galls and egg masses in peanut hairy roots overexpressing *AdEXLB8* corroborated the use of this strategy for functional characterization of root expressing candidate genes. This approach could be applicable not only for peanut–nematode interaction studies but also to other peanut root diseases, such as those caused by fungi and bacteria, being also potentially extended to other crop species displaying similar petiole-rooting competence.

## Background

Peanut (*Arachis hypogaea*) is a major food crop widely cultivated in tropical and sub-tropical regions, with Asia and Africa accounting for 90% of the world’s production followed by North America [1]. Peanut breeding programs have been hampered by the narrow genetic variability in the cultivated species, differences in ploidy and other sexual barriers that prevent crosses with their more diverse wild relatives [2]. Wild *Arachis* species are endemic to South America and constitute rich sources of new alleles to be exploited for peanut breeding [3], as tolerance to drought and salinity, and resistance to various diseases caused by fungi, bacteria, virus, and nematodes [4]. Therefore, the use of wild *Arachis* species through either interspecific hybridization [5, 6] or transgenic approaches [7] constitutes a valuable strategy for the introgression of agronomically important traits into high-yielding peanut genotypes.

Among the most important pathogens that affect peanut yield, is the root-knot nematode (RKN) *Meloidogyne arenaria* that causes large damage in Southern USA [8]. The main symptom associated with host roots infected with RKNs is the gall formation, which is a consequence of the rapid division and enlargement of plant cells induced by second-stage juveniles (J2) nematodes [9]. These enlarged cells are consequence of the development of specialized feeding structures within the vascular cylinder, called giant cells, which allow the establishment of these obligatory sedentary parasites. Once established, RKNs spend most of their life-cycle within the host root, to finally become a reproductive adult female that produces hundreds of eggs embedded in egg masses that protect them against environmental constraints [10], enabling their hatch and J2 spread across the field.

Despite the substantial increase of wild *Arachis* transcriptomic data over the past years [5, 9], the functional characterization of new candidate genes is lagging behind due to the complexity of the subsequent *in planta* validation step. The key stage of this process is to associate a biological function to a candidate gene, and also analyze possible pleiotropic effects of its overexpression or silencing in the transgenic plants. A widely adopted strategy to functionally validate a candidate gene is the use of hairy roots induced by pathogenic strains of *Agrobacterium rhizogenes* that allow the in-root assessment of a large number of candidate genes before its stable transfer into the target species, which is, in general, a more complex process [10, 11].

Hairy roots are characterized by plagiotropism, abundant mass, extensive lateral branching, vigor, and fast hormone-independent growth. As a result, transgenic hairy roots have been largely applied to molecular farming, phytoremediation, and biotransformation as well as for studying the function of genes [11, 12]. In the hairy roots, the effect of gene overexpression or silencing can be further assessed for changes in response to external stimuli, such as parasites or symbionts [13, 14]. The root–biotic interaction studies using hairy root approach have been reported under both ex vitro, as composite plants, and in vitro conditions, as axenic cultures. Composite plants are generated through the induction of transformed hairy roots on non-transformed (wild type) shoots by the inoculation, in general at the hypocotyl level, of an *A. rhizogenes* pathogenic strain. Due to its efficiency and convenience, *A. rhizogenes*-derived composite plants are considered a practical approach to study gene expression effects in ex vitro conditions, avoiding restrictive tissue culture steps. Thus, composite plants became the large-scale method of choice for the screening of candidate genes associated with in-root biology, as reported for more than one hundred plant species [11].

For cultivated peanut, hairy roots have also been explored as alternatives for functional validation of candidate genes since this species is recalcitrant to both in vitro regeneration and genetic transformation that are still laborious, space- and time-consuming processes [7]. Therefore, peanut hairy roots, as composite plants or cultivated in vitro, have been successfully applied to study genes involved in nodule formation and nitrogen fixation [15, 16], nematode interaction [17], subterranean insects [18], and drought tolerance [19]. The recent availability of peanut progenitors’ whole genomes (*A. duranensis* and *A. ipaënsis*) [3, 20], coupled with transcriptome surveys of wild species (*A. stenosperma, A. magna, A. diogoi,* and *A. duranensis*) [9, 21–23], has substantially raised the number of candidate genes involved in tolerance/resistance to abiotic and biotic stresses. As a consequence, a more efficient method is necessary for *in planta* functional validation of genes from wild species in cultivated peanut.

The present work describes a fast, simple, and efficient ex vitro methodology to generate transgenic hairy roots for functional characterization of candidate genes in peanut, particularly those involved in the *Arachis*–nematode interaction. The methodology is based on the well-known competence of peanut to produce roots spontaneously from the petiole of detached leaves. After *A. rhizogenes* inoculation, the developing transgenic hairy roots can be cultured and maintained for extended periods under ex vitro conditions to be further used for swift phenotyping [24–26]. Using this methodology, an expansin-like B gene isolated from *A. duranensis* (*AdEXLB8*) [9, 27] was successfully overexpressed in transgenic hairy roots of the nematode-susceptible peanut cultivar ‘Runner’, leading to a significant decrease in the number of RKN galls and egg masses. Although, to date, plant EXLB proteins involvement in preventing the nematode cycle completion has not yet been clarified, our results reinforce previous studies indicating that *EXLB* genes are common components in the establishment of both parasitism and symbiosis in *Arachis* and seem to play a major role in plant–microbe interactions [27–29].

The use of roots derived from detached leaves demonstrated to be a promising alternative to whole composite plants or in vitro cultured hairy roots for the screening of candidate genes in peanut. This strategy could also be extended to other crop species, which are able to produce and maintain roots from detached leaves in moist chambers for prolonged periods, such as chickpea, pigeon pea, and cotton [30].

## Methods

### Binary vector and *Agrobacterium rhizogenes* strain

**Fig. 1:**
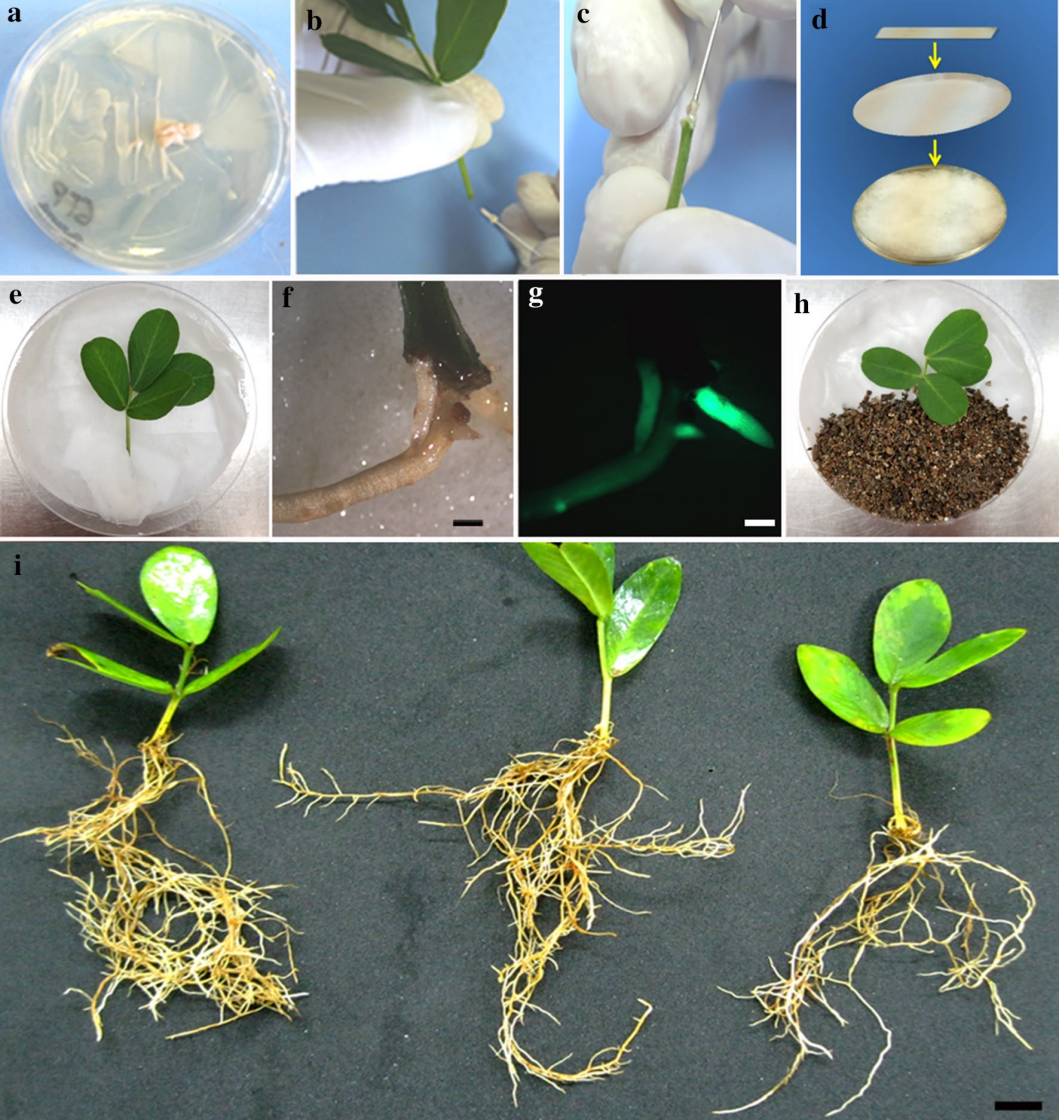
Sequential steps to obtain transgenic hairy roots from peanut detached leaves. **a** *A. rhizogenes* paste prepared for petiole inoculation. **b**, **c** Inoculation of *A. rhizogenes* paste in the peanut petiole by puncturing with a needle. **d** Diagram of the moist chamber showing the Petri dish with a cotton layer covering the moistened filter paper and a microscope slide on* top* of them. **e** Detached leaf in the moist chamber after the inoculation with *A. rhizogenes* paste and covered by wet cotton at the wounding region of the petiole. **f** Emerging hairy roots, 20 days after *A. rhizogenes* transformation, using the bright field and **g** the epifluorescent to detect eGFP. **h** Petiole covered by vermiculite after hairy roots emergence. **i** Hairy roots in peanut detached leaves, 30 days after *A. rhizogenes* transformation. *Scale bars* = 1 mm in (**f**, **g**) and = 5 mm in (**i**)

The binary vector pPZP-201BK-EGFP [17] containing the enhanced green fluorescent protein (eGFP) driven by the double 35S promoter was used as transformation control (empty vector). For gene validation, the 753 bp coding region of the RKN-resistance candidate gene *AdEXLB8* [9, 27] was cloned, under the control of the *Arabidopsis thaliana* actin 2 promoter, at the *XhoI* restriction site of pPZP-201BK-EGFP by Epoch Life Science Inc. (Texas, USA). *Agrobacterium rhizogenes* pathogenic strain ‘K599’, harboring the binary vectors pPZP-201BK-EGFP and pPZP-AdEXLB8, was inoculated directly from its −80 °C glycerol stock into a semi-solid 1.6% (w/v) agar Luria–Bertani (LB) medium containing streptomycin (100 mg/L) and kanamycin (80 mg/L) and grown for 48 h at 28 °C. A single colony was re-streaked and grown for additional 48 h. The bacterial culture was then suspended in 1 mL of liquid LB medium containing 15% glycerol (v/v). An aliquot of 200 µL was spread in the semi-solid LB medium with the proper antibiotics and grown for 24 h at 28 °C to produce a bacterial paste used for hairy root induction (Fig. 1a), essentially as described by [31].

### Hairy root induction in peanut detached leaves

The youngest fully expanded quadrifoliate leaves from *A. hypogaea* ‘Runner IAC-866’, a nematode-susceptible genotype, were harvested from 6-week-old greenhouse-grown plants, maintaining approximately 5 cm of the petiole, and immediately inoculated with a fresh bacterial paste of *A. rhizogenes* ‘K599’ engineered strains. The bacterial paste was gently introduced into the petiole of each detached leaf in a parallel position to the vascular bundle by puncturing three times on the same site (Fig. 1b, c) with a needle (23¾ gauge) (Becton, 2 Dickinson and Company, USA). After wounding, each leaf was positioned with the adaxial surface facing up onto a microscope slide, in a Petri dish containing a cotton layer covered with wet filter paper (hereafter named moist chamber) to avoid their contact with the paper (Fig. 1d, e). Inoculated petioles were maintained under moist conditions by immediately covering the wounding region with sterilized wet cotton (Fig. 1e), as previously described [32]. Detached leaves were maintained in a growth chamber at 25 ± 2 °C, with 16 h photoperiod. Whenever the first hairy root emerged (Fig. 1f, g), around 20 days after *A. rhizogenes* inoculation, the cotton over the petiole was replaced by sterilized wet vermiculite (grade 3) (Fig. 1h).

### Screening of eGFP-positive hairy roots

The eGFP expression in emerging hairy roots was verified by the presence of green fluorescence (Fig. 1f, g) using a fluorescence stereomicroscope (M205, Leica Microsystems, Wetzlar, Germany) with the GFP1 filter. Thirty days after the *A. rhizogenes* transformation, when most of the transgenic roots reached the optimal size for nematode inoculation (5 cm long; Fig. 1i), all eGFP-negative roots were excised.

### Nematode bioassay and infection assessment

*M. arenaria* J2 were isolated from roots of greenhouse-grown tomato (*Solanum lycopersicum*) plants according to [33]. Thirty-day-old hairy roots from each detached leaf (Fig. 1i) were inoculated with 1000 J2 directly onto the vermiculite.

To monitor the penetration and development of nematodes inside the hairy roots, samples were stained with the acid fuchsin according to [34], with modifications, throughout the nematode cycle. Briefly, roots were washed to remove adhering vermiculite particles and soaked in 6% (v/v) hypochlorite solution for 4 min, rewashed for 45 s and soaked in water for 15 min. Roots were transferred to boiling 0.035% (v/v) acid fuchsin solution for 2 min and rinsed in boiling water. Galls and nematodes at different stages of development could then be observed in infected roots using the stereomicroscope. The assessment of the number of egg masses in hairy roots was carried out 60 days after RKN inoculation. For that, roots were stained with phloxine B solution (0.15 g/L; w/v) for 20 min and rinsed in distilled water, prior to observation in the stereomicroscope. A *t* test determined the statistical significance (P ≤ 0.01) of the number of both galls and egg masses using the Graphpad software (http://graphpad.com/quickcalcs/ttest1/).

### Transgene expression profiling

**Fig. 2:**
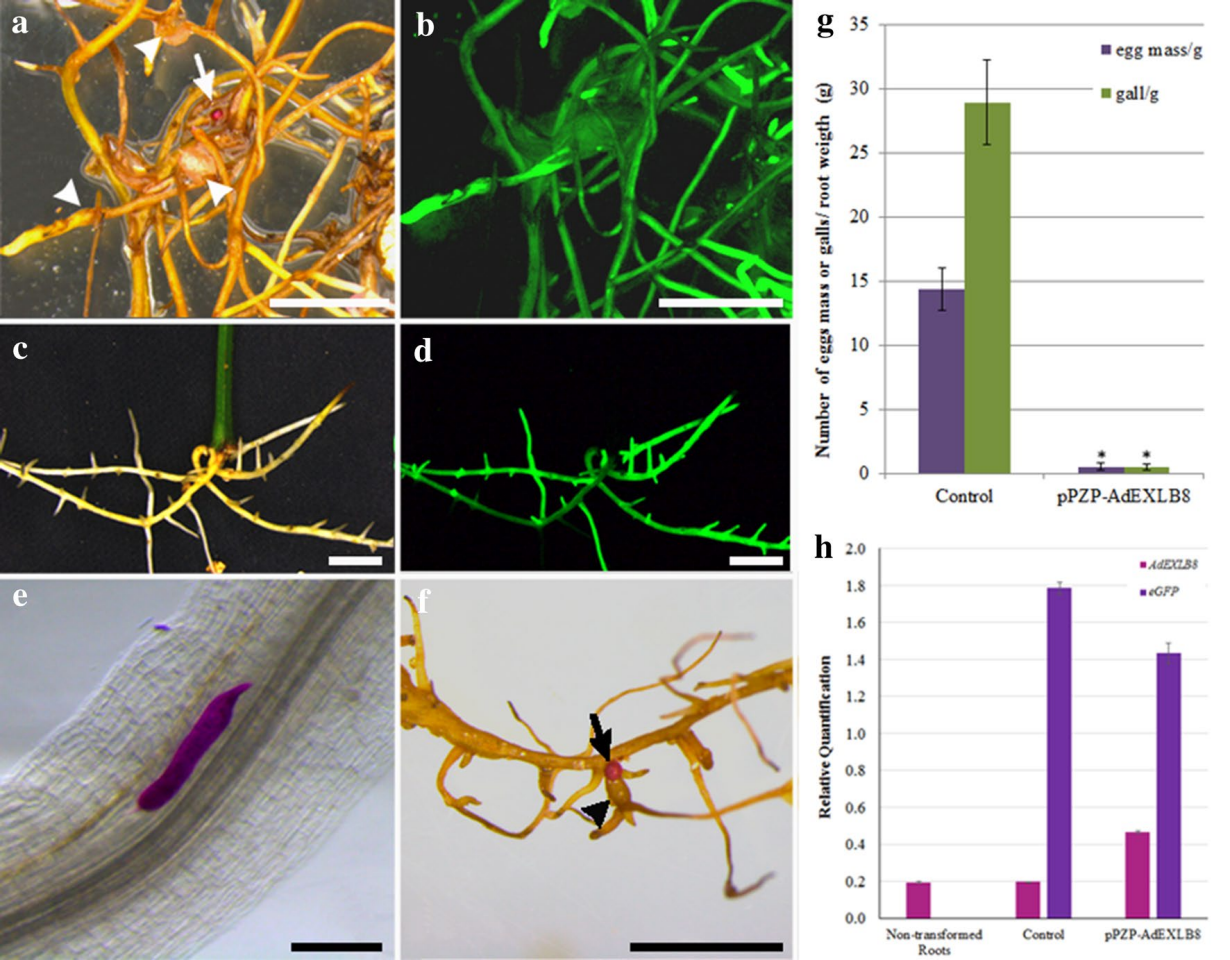
Nematode infection and development analysis in peanut hairy roots transformed with empty (control) and pPZP-AdEXLB8 vectors. **a** Control hairy roots observed in the stereomicroscope using bright field and **b** epifluorescence, 60 days after nematode inoculation. **c** pPZP-AdEXLB8 hairy roots observed in bright field and **d** epifluorescence, 60 days after nematode inoculation. **e** J4 juvenile stage of *M. arenaria* stained by acid fuchsin inside a control hairy root with the feeding site. **f** Control hairy roots with an egg mass stained with phloxine B. **g** Mean number of egg masses and galls per root gram in control and pPZP-AdEXLB8 transgenic roots. **h** Relative quantification of *AdEXLB8* and *eGFP* target genes expression in eGFP-positive hairy roots transformed with the empty (Control) and pPZP-AdEXLB8 vectors and in non-transformed (wild-type) peanut roots by qRT-PCR analysis, after normalization with the *60S* and *GAPDH* reference genes, according to the MNE procedure [36]. *Scale bars* = 5 mm in (**a**–**d**, **f**) and = 200 µm in (**e**). *Arrows* and *arrowheads* indicate egg masses and galls, respectively (**a**, **f**). *Error bars* represent the standard errors (**g**, **h**)

To evaluate transgenes (*AdEXLB8* and *eGFP*) expression, only eGFP-positive hairy roots transformed with the empty and the pPZP-AdEXLB8 binary vectors were collected at 60 days after RKN inoculation (Fig. 2a–d). As negative control, non-transformed (wild type) roots were collected from 6-week-old greenhouse-grown plants of *A. hypogaea* ‘Runner IAC-866’. Total RNA was extracted from roots using RNeasy Plant Mini kit (Qiagen, Valencia, CA), following the manufacturer’s instructions. cDNA synthesis and qRT-PCR analysis were conducted as previously described [33] using the 60S and GAPDH genes as Ref. [35] to normalize the expression of the target genes, according to the Mean Normalized Expression (MNE) procedure described by [36]. Primers used for *AdEXLB8* and *eGFP* transgenes amplification were previously designed by [21] and [37], with the amplification sizes of 149 and 104 bp, respectively. PCR amplification efficiencies for each primer pair were accessed based on the kinetics of individual reactions using the online real-time PCR Miner tool [38].

## Results and discussion

### Ex vitro hairy root induction in detached leaves petiole

A total of 50 petioles of peanut detached leaves were transformed with the *A. rhizogenes ‘*K599’ strain harboring the empty vector and 50 petioles with the pPZP-AdEXLB8 vector, in three independent experiments. Twenty days after transformation, 92 and 86% of the petioles, respectively, transformed with the vectors described above, produced at least one eGFP-positive hairy root at the wounding site. During the subsequent 10 days, new hairy roots emerged and grew vigorously with extensive lateral branches, reaching an average of five hairy roots per detached leaf, each with up to 5 cm in length (Fig. 1i). This high transformation efficiency was the result of the combination of the explant type, bacterial cell density and growth stage, *A. rhizogenes* strain, and moist chamber conditions.

Diverse types of peanut explants have been tested for *A. rhizogenes* transformation, such as petioles, epicotyls, embryonic axes, hypocotyls, and leaves, which showed different transformation efficiencies, from 14 to 91%, depending on the explant and host genotype [15–19]. Herein, the ex vitro petiole transformation efficiency obtained was similar to those observed under in vitro conditions (91%) [19]. It is worth noting that the competence of these explants to differentiate into hairy roots seems to be dependent on the peanut varietal-group (Runner, Virginia, Valencia or Spanish) [7, 39]. Previous reports suggested that peanut genotypes belonging to Valencia and Spanish are more competent to regenerate than those belonging to other varietal-groups, which displayed a recalcitrant nature [40]. Further studies showed that three *A. hypogaea* botanical varieties (*vulgaris, fastigiata*, and *peruviana*) belonging to the subspecies *fastigiata* were also able to generate composite plants and showed, respectively, 88, 81, and 72% of transformation efficiency [16]. Therefore, the choice of the peanut explant and genotype are key factors to achieve successful *A. rhizogenes* transformation [41].

The use of detached leaves as explants for *Agrobacterium* transformation seems to circumvent these issues since the natural ability of peanut to undergo adventitious rooting from this explant is genotype-independent and occurs widely in the genus *Arachis*, including wild species [24–26, 32, 42]. Moreover, the use of detached leaf explants appears to improve the efficiency of *A. rhizogenes* transformation, since the tissue wounding, which is essential for the processes of de novo root organogenesis in this type of explant [43], is also necessary for *Agrobacterium* chemotaxis response and attachment to plant cells [44, 45].

Another parameter that affects hairy root induction in peanut is the *Agrobacterium* cell density and growth stage [18, 46]. The glycerol-containing medium (paste) used in the current protocol combined both high bacterial cell density with freshly grown *Agrobacterium* culture, allowing an efficient transformation [17, 31, 47]. The inoculum paste might maintain the *Agrobacterium* exponential growth, thus avoiding bacterial overgrowth and resulting adverse effects that reduce the transformation rates, such as the production of toxic compounds at the inoculation site [48]. Besides, the inoculation of the bacterial paste inside the petiole surrounding vascular tissues through a needle might also increase the transformation efficiency since the *Agrobacterium* cells are put in direct contact with the regeneration-competent cells (procambium and vascular parenchyma) involved in root tip emergence [43]. Nevertheless, in the method described above, the bacterial cell density cannot be standardized, which might produce slight differences in transformation efficiency, as observed here using empty and pZP-AdEXLB8 vectors.

The *A. rhizogenes* ‘K599’ strain was used in our study to induce hairy roots in peanut due to its well-known hypervirulence in a wide range of plant species, including peanut [11]. Although other pathogenic wild (ATCC 15834, A4, MAFF-02-10266) and engineered (R1000, R1601, R1200) strains had also been employed to induce hairy roots in peanut [15, 17, 19, 49, 50], only the ‘K599’ wild strain was successfully used for studies on peanut–RKN interaction [17]. The *A. rhizogenes* strain type is strongly associated with the undesirable morphological variability of hairy roots, which comprises one of the main constraints to its use for functional gene studies. Hairy root phenotype (plagiotropic and fast growth, high degree of lateral branching, and profusion of root hairs) is associated with the imbalance in phytohormone levels promoted by the expression of T-DNA oncogenes and might hinder the analysis of transgene pleiotropic effects [12]. In the present study, the ‘K599’ strain induced hairy roots in peanut that displayed an attenuated phenotype (Fig. 1i) and seemed to behave as a differentiated and functional regular root organ to be further explored for in-root biology studies [51–53]. Moreover, the high phenolic content generally present in peanut tissues [54] may cause the attenuation of its hairy root phenotype, as previously reported for woody species, such as pine and gingko [55, 56].

Lastly, high humidity condition is essential for a successful formation and development of the hairy roots [17, 31, 47]. This condition can be more easily achieved inside a Petri dish, as in the small moist chamber here described, than for a whole composite plant [15, 17]. Moreover, each Petri dish accommodates up to two detached leaves, and each petiole produces between two to five independent transgenic roots. Hence, the methodology here described is practical and space-saving, facilitating large-scale analysis. Also, as an ex vitro methodology, it does not require expensive inputs and infrastructure for tissue culture, thus greatly reducing the analysis costs.

### Inoculation of peanut hairy roots with *Meloidogyne arenaria*

To validate the use of peanut hairy roots induced from detached leaves for in-root functional characterization of genes, a candidate gene for nematode-resistance, *AdEXLB8* [9, 27], was chosen to be overexpressed in transgenic hairy roots and analyzed after inoculation with the RKN *M. arenaria*. *AdEXLB8* was isolated from *A. duranensis* and belongs to the plant expansin superfamily composed by cell wall loosening proteins involved in several developmental processes and adaptive responses to environmental stimuli, including nematode infection [56].

In order to establish the bioassay for nematode inoculation in the hairy root system described above, some parameters essential for the successful RKN infection were considered, such as the type of plant substrate, moisture conditions suitable for both roots and nematodes, nematode inoculum, and the assessment of disease symptoms in the transgenic roots.

Considering that moisture is required for nematode movement and survival, overwatering or inappropriate substrate can affect nematode pathogenicity, mostly due to the lack of oxygen [57]. Here, the vermiculite was chosen as the most suitable substrate due to its high water holding capacity, inert chemical nature and adequate resistance for nematode movement. The *M. arenaria* inoculum concentration (1000 J2 in 1 mL of water per explant) was based on previous work using composite plant [17].

Using the methodology described here, transgenic roots harboring the empty vector were successfully infected by *M. arenaria*, with an average of 29 galls and 14 egg masses per root gram (Table 1; Fig. 2a, b, g). Although gall formation is a suitable indicator of the root response to nematode infection, it is not always easy to be assessed in hairy roots by acid fuchsin staining, whilst egg masses can be easily stained with phloxine B. Accordingly, the number of galls and egg masses per root gram were both used as parameters for the assessment of nematode infection in the present study. Therefore, the disease progress in the roots transformed with the empty vector was monitored in the stereomicroscope after acid fuchsin staining, which showed that *M. arenaria* was able to infect the roots, establish the feeding site (Fig. 2e), reach the maturity to produce eggs and complete its life cycle inside the host. In parallel, the production of egg masses (Fig. 2a, f) was also assessed and clearly observed in roots transformed with the empty vector, demonstrating that the hairy roots developed from the petiole of peanut detached leaves are a suitable system for screening candidate genes involved in peanut–RKN interaction. On the other hand, transgenic roots overexpressing the *AdEXLB8* gene showed a significant reduction (98%) in the number of galls and egg masses (0.05 and 0.05 per root gram, respectively; Table 1; Fig. 2c, d, g).

**Table 1:** Evaluation of *M. arenaria* infection in hairy roots derived from peanut detached leaves after transformation with the empty (Control) and pPZP-AdEXLB8 (AdELXB8) binary vectors

Detached leaf	Transgenic roots weight (g)	Number of galls	Number of egg masses	Number of galls/root gram	Number of egg masses/root gram
Control-1	0.12	8	5	66.67	41.67
Control-2	0.09	5	1	55.56	11.11
Control-3	0.34	4	2	11.76	5.88
Control-4	0.30	7	4	23.33	13.33
Control-5	0.10	3	1	30.00	10.00
Control-6	0.51	11	8	21.57	15.69
Control-7	0.26	6	4	23.08	15.38
Control-8	0.19	3	1	15.79	5.26
Control-9	0.29	4	1	13.79	3.45
Control-10	0.10	5	3	50.00	30.00
Control-11	0.25	8	7	32.00	28.00
Control-12	0.15	3	1	20.00	6.67
Control-13	0.13	5	3	38.46	23.08
Control-14	0.13	8	5	61.54	38.46
Control-15	0.07	3	2	42.86	28.57
Control-16	0.16	5	3	31.25	18.75
Control-17	0.23	3	1	13.04	4.35
Control-18	0.06	1	1	16.67	16.67
Control-19	0.18	4	3	22.22	16.67
Control-20	0.21	2	2	9.52	9.52
Control-21	0.22	4	3	18.18	13.64
Control-22	0.30	8	4	26.67	13.33
Control-23	0.25	5	2	20.00	8.00
Control-24	0.30	6	3	20.00	10.00
Control-25	0.21	5	3	23.81	14.29
Control-26	0.23	3	1	13.04	4.35
Control-27	0.34	4	4	11.76	11.76
Control-28	0.25	5	1	20.00	4.00
Control-29	0.20	12	2	60.00	10.00
Control-30	0.08	3	1	37.50	12.50
Control-31	0.23	4	3	17.39	13.04
Control-32	0.19	17	6	89.47	31.58
Control-33	0.41	5	1	12.20	2.44
Control-34	0.67	15	5	22.39	7.46
Control-35	0.28	4	1	14.29	3.57
Control-36	0.16	4	1	25.00	6.25
Control-37	0.15	6	3	40.00	20.00
Control-38	0.27	8	5	29.63	18.52
Control average	0.23	5.68	2.82	28.96	14.40
AdEXLB8-1	0.27	0	0	0	0
AdEXLB8-2	0.11	0	0	0	0
AdEXLB8-3	0.61	0	0	0	0
AdEXLB8-4	0.45	0	0	0	0
AdEXLB8-5	0.23	0	0	0	0
AdEXLB8-7	0.26	0	0	0	0
AdEXLB8-8	0.12	0	0	0	0
AdEXLB8-9	0.13	0	0	0	0
AdEXLB8-11	0.50	1	1	2	2
AdEXLB8-12	0.09	0	0	0	0
AdEXLB8-13	0.39	0	0	0	0
AdEXLB8-14	0.13	0	0	0	0
AdEXLB8-15	0.10	0	0	0	0
AdEXLB8-16	0.20	0	0	0	0
AdEXLB8-17	0.24	0	0	0	0
AdEXLB8-18	0.16	0	0	0	0
AdEXLB8-19	0.13	0	0	0	0
AdEXLB8-20	0.21	0	0	0	0
AdEXLB8-22	0.08	0	0	0	0
AdEXLB8-23	0.36	0	0	0	0
AdEXLB8-24	0.39	0	0	0	0
AdEXLB8-25	0.24	0	0	0	0
AdEXLB8-27	0.24	0	0	0	0
AdEXLB8-28	0.34	0	0	0	0
AdEXLB8-29	0.17	0	0	0	0
AdEXLB8-30	0.12	0	0	0	0
AdEXLB8-31	0.16	0	0	0	0
AdEXLB8-32	0.19	0	0	0	0
AdEXLB8-33	0.03	0	0	0	0
AdEXLB8-34	0.13	0	0	0	0
AdEXLB8-35	0.02	0	0	0	0
AdEXLB8-36	0.17	0	0	0	0
AdEXLB8-37	0.24	0	0	0	0
AdEXLB8-38	0.18	0	0	0	0
AdEXLB8-39	0.09	0	0	0	0
AdEXLB8-40	0.13	0	0	0	0
AdEXLB8-41	0.18	0	0	0	0
AdEXLB8-43	0.06	0	0	0	0
AdEXLB8 average	0.21	0.03	0.03	0.05	0.05

These results indicate that the overexpression of *AdEXLB8* decreases RKN *M. arenaria* infection in peanut, as suggested by our previous transcriptome analysis in wild *Arachis* species [9]. The overexpression of *AdEXLB8* and *eGFP* transgenes was further confirmed on pPZP-AdEXLB8 transgenic hairy roots by qRT-PCR (Fig. 2h) with primer efficiencies of 0.86 ± 0.01 and 0.91 ± 0.02, respectively. The transgene e*GFP* showed a higher expression, due to the use of the stronger double 35S promoter, than *AdEXLB8* which was driven by the actin2 promoter from *A. thaliana* [17]. The *AdEXLB8* transgene expression levels in pPZP-AdEXLB8 hairy roots are 2.4 times higher than that of the endogenous peanut *AhrunnerEXLB8* gene in the non-transformed (wild-type) peanut roots and in the eGFP-positive hairy roots transformed with the empty vector (Fig. 2h). To further support current results, the overexpression of *AdEXLB8* in soybean (*Glycine max*) composite plants infected with *M. javanica* also led to the reduction of the gall number in the susceptible genotype [27]. However, the biological mechanisms by which the overexpression of this cell wall-loosening and non-enzymatic protein contributes to RKN-resistance in peanut and soybean must be pursued.

## Conclusions

This is the first study that describes a highly efficient ex vitro transformation methodology for detached leaves in peanut. This practical, fast and low-cost methodology allows high-throughput analysis of in-root expressed candidate genes. As an ex vitro methodology, the detached leaves in Petri dishes requires little space, and greatly reduces the cost of phenotyping. Moreover, this strategy is suitable for plant–nematode interaction studies, since it avoids critical steps of nematode sterilization and the complexity of maintaining an axenic system comprised by three organisms: plant/nematode/bacteria. Besides, it can also be applied to in-root biology studies of a broader spectrum of crop species. This methodology can be promptly applied for candidate gene validation for nematode resistance and other peanut root pathogens, as shown here by the overexpression of *AdEXLB8* gene, conferring resistance to *M. arenaria* in transgenic peanut hairy roots.

## Abbreviations

RKN: root-knot nematode
eGFP: enhanced green fluorescent protein
LB: Luria–Bertani
J2: second-stage juveniles
